# European Network of Bipolar Research Expert Centre (ENBREC): a network to foster research and promote innovative care

**DOI:** 10.1186/2194-7511-1-2

**Published:** 2013-04-17

**Authors:** Chantal Henry, Ole A Andreassen, Angelo Barbato, Jacques Demotes-Mainard, Guy Goodwin, Marion Leboyer, Eduard Vieta, Willem A Nolen, Lars Vedel Kessing, Jan Scott, Michael Bauer, The ENBREC study group

**Affiliations:** 1 Université Paris-Est, UMR_S955, UPEC, F-94000 Créteil, France; 2 Inserm, U955, Equipe Psychiatrie Génétique, F-94000 Créteil, France; 3 AP-HP, Hôpital H. Mondor - A. Chenevier, Pôle de Psychiatrie, F-94000 Créteil, France; 4 Fondation FondaMental, fondation de coopération scientifique, F-94000 Créteil, France; 5 KG Jebsen Centre for Psychosis Research, Division of Mental Health and Addiction, Oslo University Hospital & Institute of Clinical Medicine, University of Oslo, Oslo, Norway; 6 Laboratory of Epidemiology and Social Psychiatry, Mario Negri Institute for Pharmacological Research, Milan, Italy; 7 European Clinical Research Infrastructures Network (ECRIN), INSERM, Paris, France; 8 University Department of Psychiatry, University of Oxford, Oxford, UK; 9 Institute of Neuroscience, Hospital Clinic, University of Barcelona, IDIBAPS, CIBERSAM, ENBREC, Barcelona, Catalonia Spain; 10 Department of Psychiatry, University Medical Center Groningen, University of Groningen, Groningen, The Netherlands; 11 Psychiatric Center Copenhagen Department, Faculty of Health Sciences, University of Copenhagen, O 6233, Blegdamsvej 9, 2100 Copenhagen, Denmark; 12 Academic Psychiatry, Institute of Neuroscience, Newcastle University, Newcastle-upon-Tyne, UK; 13 Department of Psychiatry and Psychotherapy, University Hospital Carl Gustav Carus, Technische Universität, Dresden, Germany; 14 ENBREC, European Commission, Smith Square, London, UK

**Keywords:** Bipolar disorder, Clinical practice, Research networks, Standards of care

## Abstract

Bipolar disorders rank as one of the most disabling illnesses in working age adults worldwide. Despite this, the quality of care offered to patients with this disorder is suboptimal, largely due to limitations in our understanding of the pathology. Improving this scenario requires the development of a critical mass of expertise and multicentre collaborative projects. Within the framework of the European FP7 programme, we developed a European Network of Bipolar Research Expert Centres (ENBREC) designed specifically to facilitate EU-wide studies. ENBREC provides an integrated support structure facilitating research on disease mechanisms and clinical outcomes across six European countries (France, Germany, Italy, Norway, Spain and the UK). The centres are adopting a standardised clinical assessment that explores multiple aspects of bipolar disorder through a structured evaluation designed to inform clinical decision-making as well as being applicable to research. Reliable, established measures have been prioritised, and instruments have been translated and validated when necessary. An electronic healthcare record and monitoring system (e-ENBREC©) has been developed to collate the data. Protocols to conduct multicentre clinical observational studies and joint studies on cognitive function, biomarkers, genetics, and neuroimaging are in progress; a pilot study has been completed on strategies for routine implementation of psycho-education. The network demonstrates ‘proof of principle’ that expert centres across Europe can collaborate on a wide range of basic science and clinical programmes using shared protocols. This paper is to describe the network and how it aims to improve the quality and effectiveness of research in a neglected priority area.

## Review

### Background

Bipolar disorders (BD) are characterised by recurrent manic and depressive episodes that usually commence in early adulthood and affect 1% to 4% of the general population (Merikangas et al. 2007). According to the World Health Organization study on global burden of disease, BD are ranked sixth amongst the most disabling illnesses in working age adults worldwide (above schizophrenia which is ranked eighth) (Lopez and Murray 1998) - findings that are reinforced by the recent European study on the burden of mental health (Olesen et al. 2012).

Despite its high prevalence, BD is often unrecognised or misdiagnosed leading to inappropriate or delayed treatments, with significant and devastating health and social consequences (Baca-Garcia et al. 2007; Hirschfeld et al. 2003; Scott and Leboyer 2012). Even when the diagnosis is established, it is clear that the management of BD is a major challenge, and surveys confirm that suboptimal treatment is a common concern across Europe (Scott et al. 2006). The significant disease burden attributable to BD is amplified by additional, often multiple psychiatric and physical comorbidities, and premature mortality (Leboyer and Kupfer 2010). The reduced life expectancy in BD due to medical comorbidity and adverse lifestyles is about 10 years for men and 11 for women (Chang et al. 2011). Furthermore, rates of completed suicide have generally been estimated to be between 10% and 20% (Müller-Oerlinghausen et al. 2002). Even if recent studies are more optimistic, rates in BD exceed unipolar depression and schizophrenia (Bostwick and Pankratz 2000; Dutta et al. 2007).

Whilst adherence to clinical practice guideline recommendations can improve outcome for patients (Goodwin et al. 2009; Nivoli et al. 2011a, b), many of the algorithms for BD patients are derived from efficacy data from randomised controlled clinical trials (RCTs). Such RCTs usually recruit homogeneous samples of patients who will represent only about 20% of the BD population treated in day-to-day clinical practice (Vieta and Cruz 2012). Also, guidelines are not consistent in their recommendations, differing in their advice on when to use adjunctive psychological therapies or which type of therapy to offer to different patient subpopulations (Henry et al. 2011). As a consequence, clinicians often find it difficult to systematically apply recommendations to individual cases. For example, many guidelines suggest monotherapy as maintenance treatment (based on findings from RCTs); the clinical reality is that many patients receive polypharmacy for mood stabilisation (Frye et al. 2000; Wolfsperger et al. 2007). Other strategies, extending beyond the use of guidelines, therefore need to be examined, especially because the evidence suggests that efforts made to spread good clinical practice invariably result in gains for patients. For example, Bauer et al. (2009) have shown that promoting systematic assessments and offering local support to clinicians working with BD improve patient outcomes. Similar national initiatives are being undertaken, e.g. the French BD network developed by *Fondation FondaMental* (Henry et al. 2011) and the CIBERSAM in Spain (Vieta 2011). However, to date, there have been few attempts to facilitate an international programme on the translation of research knowledge into evidence-based clinical practice. This is especially likely to be beneficial in multifaceted disorders such as BD, where a ‘personalised medicine’ approach represents the best way to take into account the diversity of clinical presentation, including the frequent presence of comorbidities, and the range and optimal combinations of pharmacological and psychosocial treatments (Scott 2011).

The nature and complexity of BD mean that successful translational research requires the integration of advances in clinical and basic science to develop targeted treatments that are more specific for BD. However, improving research in the field of severe and complex disorders increasingly requires the development of a critical mass of expertise through broadly based collaborative networks. Multicentre projects maximise the likelihood of recruitment of large clinical cohorts that reflect the heterogeneity of the disorder and facilitate cross-national epidemiological studies, as well as providing subgroups with adequate statistical power to explore pathophysiological and gene-environment interactions and to conduct clinical trials. This type of research is only usually achievable through the development and implementation of shared research protocols across a large number of centres (Vieta et al. 2011).

In order to disseminate systematic clinical assessment and high quality treatment protocols and to foster research to improve the management of BD and to develop a better understanding of the mechanisms underlying this complex condition, we have developed a network of bipolar expert centre at a European level: European Network of Bipolar Research Expert Centre (ENBREC, http://www.enbrec.eu). This network was set up via FP7 funding but is maintained through the support from the European College of Neuropsychopharmacology (ECNP) network initiative (ECNP-NI). In this paper, we describe the development of the ENBREC network and the common, cross-national clinical and research tools we are introducing.

## Results and discussion

In 2009, the European Union resolution on Mental Health (EU parliament A6-0034-2009) highlighted the need to develop comprehensive and integrated mental health strategies in Europe, such as cohort studies. As we live in a globally competitive environment, it is fundamental for Europe's success that high level research is viewed as a priority. This will demand joint responses from member states and between-country collaborations so that expertise and experience can be shared, and benefits can be rapidly disseminated on a Europe-wide basis. The EU resolution also acknowledges that the only way to diminish the cost and burden of mental disorders in the long-term is to invest resources in research to improve early diagnosis, develop innovative treatments, and to attempt to identify individuals at high risk of developing mental disorders with the ultimate goal of implementing prevention strategies. The ECNP is also concerned to support the development of independent collaborative international research networks of basic scientists and practising clinicians within Europe, and has established the ECNP-NI to help meet this goal. The aims and activities of its component networks are determined by the experience and expertise of the participating members, but each has the goal of extending current understanding of the causes and treatment of central nervous system disorders, thereby contributing to improvements in clinical outcomes and reducing the societal burden of mental and neurodegenerative disorders. Given the aspirations of these organisations, it is understandable that both have supported the development and activities of ENBREC. There is a need for a coordinated approach to developing patient cohorts for the more prevalent mental disorders representing major mental health challenges such as BD. In this context, integrated research programmes are clearly useful as clinical, epidemiological and cognitive data can be combined with biological studies such as brain imaging, genetics or neurobiology.

Expert centres provide a unified setting for high-quality care and research (Henry et al. 2011; Vieta 2011). Such expert centres have been shown to improve outcome of bipolar disorder compared to standard care (Kessing et al. 2013).

We believe that embedding cohort studies within our collaborative network is the most efficient approach to obtain cross-sectional and longitudinal information about BD. The clinical setting of participating centres will also allow speedy implementation of improved diagnostic and assessment strategies. Whilst a deeper knowledge of the pathophysiology of mental health is required to improve the management of diseases and the development of new treatments, the programme will strengthen translational research delivering a new, broad, comprehensive understanding of BD from basic science to biomedical research. The ultimate clinical aim of ENBREC is to develop personalised medicine, aspiring to assess each patient using a spectrum of behavioural and neurobiological measures in order to define the best therapeutic strategies relevant to a particular profile of the disorder. Implementing prevention, early diagnosis and personalised interventions will allow ENBREC to demonstrate ways to improve personal and economic outcomes of mental health care.

This network offers a ‘proof of principle’ that expert centres across Europe can undertake collaborative studies using shared assessment protocols. The purpose of this network is to improve the quality and efficiency of research in the neglected priority area of mental health, specifically mood disorder.

This type of collaborative project can have a positive impact on attitudes and can bring together individuals who actively seek opportunities for collaboration. This project both supports existing collaborations and helps create new ones. This is of utmost importance for general psychiatry and for BD research. In spite of the major burden of disease for the society, mental health research as a whole (and BD research in particular) is currently hampered by several gaps. These include a gap in structuring due to its cross-disciplinary nature, encompassing a broad range of disciplines from psychology to molecular biology, a gap in funding, as a result of the poor structuring of mental health research at the national and European level, and a gap in met and unmet clinical needs, because research priorities are not always translated into clinical advances in a timely way or do not adequately reflect the priorities of day-to-day practice.

## Methods

### Resources and goals

Following a successful application to the ‘Support Action’ call of the European FP7 programme in 2009, we set about developing the infrastructure for ENBREC, a disease-oriented EU-wide network designed to foster multinational collaboration among centres with expertise in the clinical management of and/or research in BD. The programme has now evolved into one of the networks supported by the ECNP. For Europe, the added value of ENBREC is that expertise in different research fields including epidemiology, genetics and clinical trial design is shared across centres and that leading researchers in the field of BD gain early access to large patient cohorts, improving the clinical representativeness of research samples and enhancing the external validity and statistical power of proposed studies. A core goal of ENBREC is to establish connections between leading centres in the field and to foster the development of or support existing national networks, with some of the national centres being incorporated into the Europe-wide network.

From a clinical perspective, the goal of ENBREC is to develop assessment tools and procedures to improve the diagnosis and management of BD. In addition, the cross-centre links enhance clinical investigations and observational studies, which benefit from the pooling of relevant resources including access to patients. To achieve this, there was first a need for centres to work together to develop a common assessment protocol and to develop mechanisms for efficient sharing of clinical data and biomaterial.

A separate but linked goal for the network is to disseminate clinical and basic research ideas to others working in BD in Europe and elsewhere and to produce consensus papers that highlight how to translate research findings into improved health care and clinical practice. The network also offers an unrivalled opportunity to undertake joint training of the next generation of researchers and clinicians wishing to develop expertise in BD, and joint applications for postgraduate training have been produced.

### Participants and organisation

Six European countries are currently involved in the network (France, Germany, Italy, Norway, Spain and the UK) with a maximum of two centres per country. The coordinator (Chantal Henry, France) is in charge for day-to-day management of the ENBREC network, whilst the ENBREC project committee, composed of one representative for each country, determines the overall direction and work of the network. The programme of work is divided into work-packages (WPs) organised by a WP leader (Table 1).

**Table 1:** **Work packages supported by the ENBREC network**

Work package	Scope	Lead
WP1	Management of the project	Chantal Henry, France
WP2	Developing common tools for diagnosis and multinational cohort follow-up	Eduard Vieta, Spain
WP3	Developing common tools for neurocognitive assessment	Guy Goodwin, UK
WP4	Assessment of common biomarkers and genetic markers	Marion Leboyer, France
WP5	Development of standards for imaging	Ole Andreassen, Norway
WP6	Treatment optimization, definition of subgroups of responders	Michael Bauer, Germany
WP7	Supporting multinational clinical research and data management	Jacques Demotes, France
WP8	Education, information, dissemination, translation of research outcomes into healthcare	Angelo Barbato, Italy
WP9a	Pan-European educational programme - ‘Improving the identification of BD II disorders’	Jan Scott, UK
WP9b	Extension to new countries and within the countries	Jacques Demotes, France

### A standardised assessment package

All centres are in the process of adopting the same evaluation package, with many having already achieved full implementation (Henry et al. 2011). The package comprises a wide-ranging psychobiosocial assessment that systematically explores all aspects of BD including clinical presentation, personal history and factors that potentially influence the course and outcome of BD. The measures provide a high quality, structured evaluation that can inform clinical decision making, but the tools selected are also relevant and applicable to research projects. Valid, reliable and established observer- and self-rating scales were chosen in preference to idiosyncratic or untested measures. Where appropriate, assessment tools were translated into different languages, and validation studies have been undertaken as required.

#### Clinical data

Agreement was reached on a core set of pre-existing rating scales that will be used for establishing diagnosis, evaluating symptoms, assessing functioning and collecting patient-reported data (Table 2). As well as established and nationally validated measures, some new instruments are also being ‘trialled’ in selected centres. For example, in Spain, a dimensional assessment for BD, the Dimensional Assessment of Mental Nosology for DSM, has been developed to explore trans-diagnostic aspects of symptom evaluation (see Vieta and Phillips 2007).

**Table 2:** **Core clinical rating scales**

Rating scales	Reference
**Psychiatric diagnosis**	
Structured Clinical Interview for DSM-IV Axis I Disorders	First et al. 2002
or Mini-International Neuropsychiatric Interview	Sheehan et al. 1998
Clinical Global Impression-Bipolar Version - Modified	Vieta Pascual et al. 2002
Montgomery-Åsberg Depression Rating Scale	Montgomery and Åsberg 1979
Young Mania Rating Scale	Young et al. 1978
**Functioning**	
Global Assessment of Functioning Scale	American Psychiatric Association 2000
Functioning Assessment Short Test	Rosa et al. 2007
**Self-rating scales for patients**	
State-Trait Anxiety Inventory	Marteau and Bekker 1992
Altman Self-Rating Scale for Mania	Altman et al. 1997
Quick Inventory of Depressive Symptomatology	Rush et al. 2003

Polarity, duration and severity are carefully evaluated for the current presentation whether the symptoms reach syndromal criteria or are or subsyndromal. This information is supplemented by data on the initial clinical presentation (including age of onset of the first symptoms, age of onset and polarity of first mood episode, age of first psychotropic treatment and age at first hospitalisation). Details of the total number of manic and total number of depressive episodes, number of hospitalisations, the presence or absence of rapid cycling, and occurrence of any postpartum episodes and predominant polarity (Colom et al. 2006) are also recorded. Psychiatric and medical comorbidities are also recorded, as are any history of suicide attempts and family history of mental disorders.

Past and current pharmacological treatments with psychotropic drugs are documented in the evaluation, with class of drug, dosage and duration of each treatment. Adherence to treatment (ranked globally as good, moderate or poor) is also recorded, as well as the degree of improvement with current treatment. Other previous treatments are also documented, including details of physical treatments, (such as electroconvulsive therapy or trans-cranial magnetic stimulation) and psychosocial interventions (such as cognitive-behavioural therapy, interpersonal therapy and individual or family psycho-education). Physical measurements at inclusion include metrics (height, weight and abdominal perimeter), vital signs (blood pressure and heart rate) and a full blood and biochemistry work-up.

#### Neurocognitive data

The major issues for the network with regard to the routine use of neurocognitive testing were the lengthy and time-consuming nature of assessments and the lack of feasibility of their use in a multidimensional assessment package (Goodwin et al. 2008). Therefore, ENBREC members who are experts on neurocognition reviewed the current literature on BD and cognitive impairment and, from this, devised a minimum cognitive battery for inclusion in e-ENBREC. The selected tests include a verbal learning and memory assessment (based on California Verbal Learning Test), Forward and Reverse Digit Span (from WAIS-R Digit Span), and Trail Making Test A and B. These tests are expected to provide robust measures of cognitive functioning in BD patients across several domains and will allow deficits to be identified and monitored over time as the illness progresses and/or new treatments are developed. ENBREC findings suggest a relationship between cognitive impairment and treatment adherence (Martinez-Aran et al. 2009).

### Data collection: electronic records and shared database

It is important for multisite collaborations that involve simultaneous collation of assessments to have efficient procedures for data capture that do not overburden clinicians or researchers but that maximise the likelihood of complete and accurate data entry. To help in this process, a specific case report form was produced, incorporating all the items in the standardised assessment package which was then translated into the languages of participating countries for local use.

A web-based application, e-ENBREC©, has also been developed to collate assessment data for clinical monitoring and research purposes. Access to the system is carefully regulated, and approval has to be obtained from the committee in charge of the safety of computerised databases in each country. To optimise data entry and retrieval, free text input has been minimised, and drop-down lists and other approaches leading to standardised inputs have been chosen whenever possible. The XML format is used to transfer data from e-ENBREC©(European network of bipolar research) expert centre into an anonymised common database for research purpose.

### Specific research projects

In addition to the core clinical and cognitive datasets being documented for clinical cases, specific data sets are being developed for research or diagnostic purposes in participating countries. These can be linked to the clinical database in order to allow network-wide evaluation and, thus, increase the statistical power of basic science studies. Examples of these include:

- Collection of DNA The aim of this project is to collect samples of biomaterial to perform genetic and biomarker studies. This will enable the collection of large DNA samples from patients who have been well characterised from a clinical, cognitive and, potentially, imaging perspective. The analysis of more homogenous patient groups with well-specified clinical features should facilitate the identification of potential genetic markers of BD. Existing procedures already allow ENBREC partners to exchange biological material. The Material Transfer Agreement has already been used for this purpose by ENBREC partners.
- Brain imaging Brain imaging techniques have revolutionised the understanding of the human brain, with potential to identify brain pathology underlying psychiatric disorders, including bipolar disorders (Rimol et al. 2010). In addition, it has the potential to become a tool for early identification, subgrouping, disease monitoring and treatment stratification. The overall aim of the ENBREC project is to develop a common MRI protocol which allows pooling of data acquired with standard clinical MRI scanners that can be used in multisite studies of bipolar disorders. Structural MRI studies usually provide global estimates of gray or white matter volume changes, or a small number of regions of interest. Recent advances in structural imaging now allow for a more comprehensive evaluation of brain changes by providing continuous maps of cortical thickness and surface area, subcortical volumes and measures of white matter microstructure throughout the brain (Fischl and Dale 2000). White matter can now be quantified using diffusion tensor imaging. It will be important to cross this information with cognitive data in large cohorts of patients in order to address any structural features underlying the pathology (Forcada et al. 2011). In the future and if funding permits, it is hoped to extend the imaging protocols of functional MRI.

### Promoting innovative care and treatment protocols

A significant number of patients with BD, who achieve neither clinical remission nor functional recovery despite high levels of medication adherence (Rosa et al. 2011) and recurrence rates following a manic episode, are about 40% to 60% even when receiving maintenance drug therapy (Gitlin et al. 1995). Such data highlight the need to address nonpharmacological factors in order to improve outcome and to encourage patients to take an active role in the management of their illness and collaborate in the care process. Surveys of patient preferences reveal that there is a strong wish by patients with BD to undertake self-help and participate in psychological interventions (Pontin et al. 2009).

Evidence from research supports the efficacy and likely effectiveness of a number of psychosocial treatments, including group psycho-education, family-focused therapy, interpersonal and social rhythm therapy, and cognitive behaviour therapy (Miklowitz and Scott 2009; Vieta et al. 2009). The lower cost and potential ease of dissemination of group psycho-education suggest that its use as a first-line approach warrants exploration. Research by members of ENBREC suggests that a number of elements should ideally be included in a basic psycho-education package. The key elements of the package and options for delivery such as 11 sessions of group psycho-education supplemented by a DVD, and an abbreviated psycho-education package, are currently being explored by ENBREC centres. The recommended components of a basic psycho-education package are the following:

- Use of a mood diary and life-event charting to monitor mood patterns and effectiveness of intervention
- Awareness of medication effects and improvement of decision-making skills on drug treatment in a collaborative way
- Identification of early warning signs
- Encouragement of structured routines and healthy lifestyles
- Stabilisation of sleep/wake cycles
- Emotional self-regulation and social skills
- Improvement of communication skills
- Acquisition of balanced attitudes towards the self in relation to the illness
- Reduction of self-stigmatisation

Despite promising research findings, dissemination remains an unresolved issue. Within the ENBREC network, a working group has been mandated to recommend how best to extend psycho-education techniques that have been shown to be effective into every level of everyday psychiatric care.

### Sustainability of the network: links maintained beyond initial phase of ENBREC

By the time the FP7 funding ended (June 2011), ENBREC members had engaged in and continue to be involved in several joint research projects at a Europe-wide level. The ongoing support of ECNP-NI is also vital to the continuation of the collaborative group. The sustainability of the network is secured because the centres involved in the collaboration are all clinically active, offering expert care and treatment of BD. Resources are provided within each country to ensure continuity of clinical care. The common clinical and cognitive assessment and the follow-up protocol chosen for ENBREC represent a basic, but systematic and comprehensive assessment of cases which allows the expert centres to provide high-quality advice on diagnosis and treatment to the referrers and patients in each participating country. At the same time, a major potential barrier to cross-national research has been overcome, allowing easier implementation of new basic science and clinical research studies in the future. The closely identified research group will also be in a strong position to apply for multicentre research grants and potentially represents an attractive option to international industrial partners who, for example, wish to plan and execute pilot studies or pivotal randomised controlled clinical trials. Industry-funded unrestricted educational grants are also being explored to help support the training initiatives that have grown out of the research programme.

## Conclusions

Currently, there is no equivalent multisite collaboration in the field of BD in Europe, which is able to conduct research within an organised disease-oriented clinical network, including multidisciplinary and trans-cultural approaches and with a long-term perspective. The network will provide useful clinical data from a European sample of patients. A major strength of the network is its capacity to develop research from basic science to psychosocial research programmes. It also contributes to the ECNP-NI portfolio. In the near future, the efforts of multiple stakeholders, promoters and funding agencies will need to converge into more efficient and coordinated initiatives under the umbrella and support of ‘networks of networks’, which should avoid redundancies and inefficiencies in research. ENBREC may be the seed of such a Network in the field of BD.

## Authors’ information

ENBREC study Group: The authors plus *France*: B Étain, C Rémond, S Jamain, F Bellivier; *Norway*: G Morken, *Italy*: B D’Avanzo, A Parabiaghi, F Rapisarda, M Vallarino; *United Kingdom*: C Bourne, JR Geddes*; Spain*: C Torrent, CM Bonnin; *Germany*: A Pfennig, P Ritter, European Network Initiative : N. van der Wee and Patrice Boyer
